# Auto-HMM-LMF: feature selection based method for prediction of drug response via autoencoder and hidden Markov model

**DOI:** 10.1186/s12859-021-03974-3

**Published:** 2021-01-28

**Authors:** Akram Emdadi, Changiz Eslahchi

**Affiliations:** 1 Department of Computer and Data Sciences, Faculty of Mathematical Sciences, Shahid Beheshti University, Tehran, Iran; 2 School of Biological Sciences, Institute for Research in Fundamental Sciences (IPM), 193955746 Tehran, Iran

**Keywords:** Cancer, Drug response, Autoencoder, Hidden Markov model, Matrix factorization, Personalized treatment

## Abstract

**Background:**

Predicting the response of cancer cell lines to specific drugs is an essential problem in personalized medicine. Since drug response is closely associated with genomic information in cancer cells, some large panels of several hundred human cancer cell lines are organized with genomic and pharmacogenomic data. Although several methods have been developed to predict the drug response, there are many challenges in achieving accurate predictions. This study proposes a novel feature selection-based method, named Auto-HMM-LMF, to predict cell line-drug associations accurately. Because of the vast dimensions of the feature space for predicting the drug response, Auto-HMM-LMF focuses on the feature selection issue for exploiting a subset of inputs with a significant contribution.

**Results:**

This research introduces a novel method for feature selection of mutation data based on signature assignments and hidden Markov models. Also, we use the autoencoder models for feature selection of gene expression and copy number variation data. After selecting features, the logistic matrix factorization model is applied to predict drug response values. Besides, by comparing to one of the most powerful feature selection methods, the ensemble feature selection method (EFS), we showed that the performance of the predictive model based on selected features introduced in this paper is much better for drug response prediction. Two datasets, the Genomics of Drug Sensitivity in Cancer (GDSC) and Cancer Cell Line Encyclopedia (CCLE) are used to indicate the efficiency of the proposed method across unseen patient cell-line. Evaluation of the proposed model showed that Auto-HMM-LMF could improve the accuracy of the results of the state-of-the-art algorithms, and it can find useful features for the logistic matrix factorization method.

**Conclusions:**

We depicted an application of Auto-HMM-LMF in exploring the new candidate drugs for head and neck cancer that showed the proposed method is useful in drug repositioning and personalized medicine. The source code of Auto-HMM-LMF method is available in https://github.com/emdadi/Auto-HMM-LMF.

## Background

Computational models for personalized medicine make it possible to understand cancer cell lines on the basis of genomic information. This knowledge makes it possible to recommend individualized therapies to patients with different types of cancer by measuring drug responses. Many effective anticancer drugs have already been developed for each cancer type, such as breast cancer, lung cancer, ovary cancer and Brain cancer. For example, docetaxel, paclitaxel, carboplatin, cisplatin, vinorelbine and eribulin are just a few examples of drugs used to treat breast cancer. Since drug response to cancer treatment depends on multiple factors such as the patient's genomic profile, this process is a complicated problem in cancer treatment. These challenges have generated large-scale experiments on human cancer cell lines and various anticancer drugs. For instance, two datasets Genomics of Drug Sensitivity in Cancer [1] (GDSC) and Cancer Cell Line Encyclopedia [2] (CCLE) are created based on drug sensitivity data of established anticancer drugs against diverse cancer cell lines. The various genetic features for the panels of cancer cell lines, such as gene expression profile, copy number alteration, single nucleotide mutation and methylation data, have been provided. By using these databases, machine learning algorithms are increasingly being applied to the predictions of drug responses by integrating data from different sources in a statistically meaningful way.

Several recommender system-based models were proposed for predicting drug response. Wang et al. adopted a similarity-regularized matrix factorization (SRMF) method to predict anticancer drug responses of cell lines using the gene expression profile in cell lines and drugs’ chemical structures. They indicated that rapamycin (an mTOR inhibitor) could be a new therapeutic agent for non-small cell lung cancer [3]. Suphavilai et al. developed a model, termed Cancer Drug Response prediction using a Recommender System (CaDRReS), to learn projections for drugs and cell lines into a latent space. Also, they demonstrated how to explore drug mechanisms and drug-pathway associations using the achieved features [4]. Emdadi et al. proposed DSPLMF method based on a logistic matrix factorization approach for predicting anticancer drug response. DSPLMF focuses on discovering significant features and latent vectors of cell lines and drugs for computing the probability of the cell lines are sensitive to drugs. They used the obtained latent vectors to identify subtypes of the cancer cell line and drug-pathway associations [5].

Identifying the optimal subset of features from many genetic candidate features is a crucial issue for classification models for predicting drug response. Thus, a large number of algorithms have proposed using different approaches for feature selection. Xu et al. proposed AutoBorutaRF method based on feature selection for predicting drug response. This method first built an autoencoder network, and it used Boruta algorithm [6] to select important features for applying the RandomForest classifier to predict drug response [7]. Dong et al. proposed a model termed Support Vector Machine Recursive Feature Elimination (SVM-RFE), which used a wrapper method using a recursive feature selection and SVM classifier to predict drug response [8].

This study presents a feature selection-based method for drug response prediction, named Auto-HMM-LMF, to efficiently predict cell line-drug associations. Gene expression profile, copy number alteration, single-nucleotide mutation, tissue type information of the cell line, and drugs’ chemical structure information were incorporated. Two strategies based on autoencoder and hidden Markov model-multinomial mixture model are used for selecting the essential features of input information. The autoencoder networks are applied on gene expression profile, copy number alteration data. Also, hidden Markov model and multinomial mixture model are applied on mutation data. A proper evaluation of the Auto-HMM-LMF method using tenfold cross-validation was carried out to compare it with the state-of-the-art methods. Results show its performance is superior for the tested data sets. Also, by comparing to the ensemble feature selection method (EFS), we showed that two considered strategies for feature selection in the Auto-HMM-LMF method could select proper features that significantly improved the prediction result.

## Methods

**Fig. 1:**
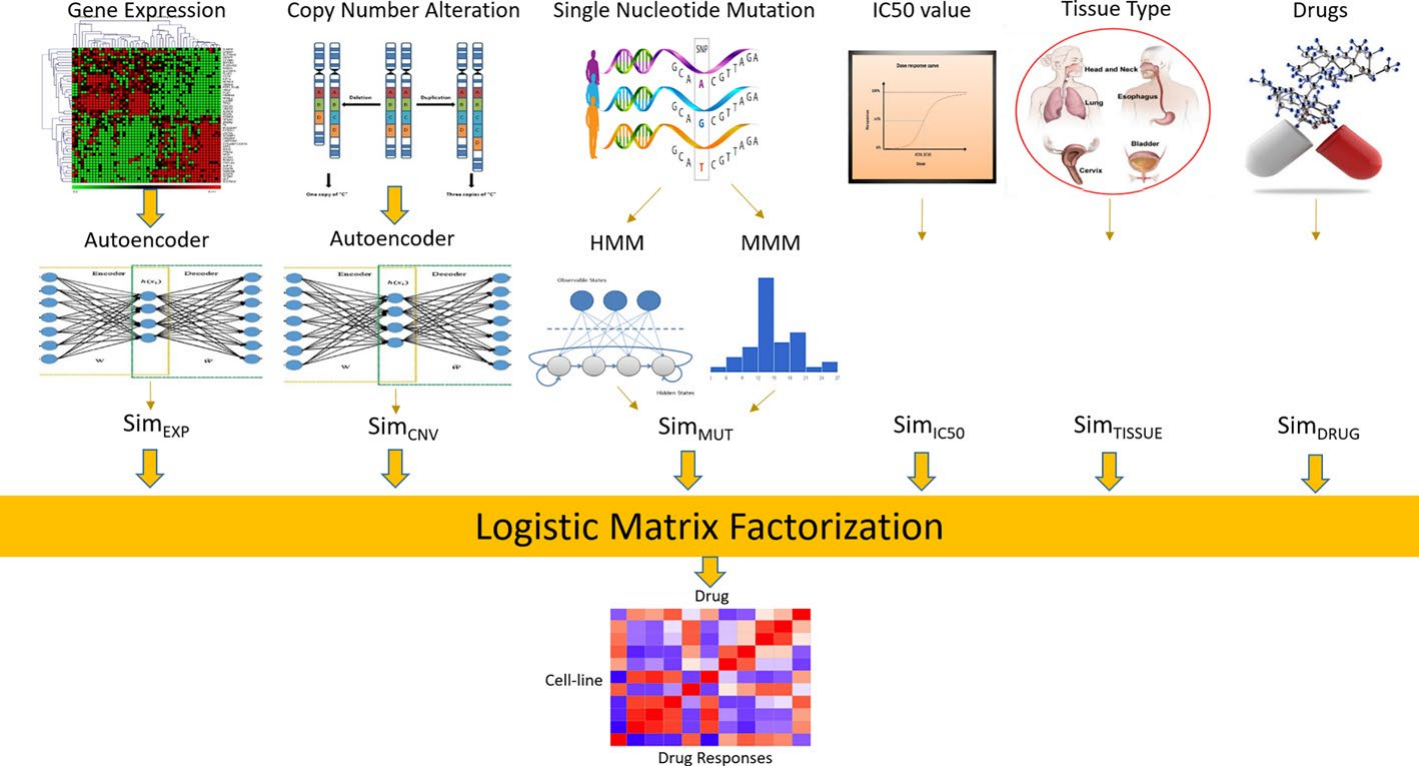
Schematic overview of Auto-HMM-LMF. Two feature selection models based on autoencoder are used for gene expression, and copy number alteration information. A novel feature selection method based on the hidden Markov model (HMM) and multinomial mixture model (MMM) is applied for single-nucleotide mutation data. In the next step, the five similarities between each pair of cell lines and the similarity between each pair of drugs are defined. For the prediction of the drug response of the cell lines across the drugs, the logistic matrix factorization method is applied

This paper proposes a novel method (Auto-HMM-LMF) to efficiently predict cell line-drug associations by combining and effectively using feature selection approaches. The main scheme of the Auto-HMM-LMF algorithm is represented in Fig. 1. In the first step, two strategies for selecting the important features of input data are used. A feature selection approach based on autoencoder networks is applied to the gene expression profile of cell lines, and the similarity matrix (Sim_EXP_) is constructed using selected features. Similarly, the similarity matrix (Sim_CNV_) is created using the selected feature by applying the autoencoder feature selection method on copy number alteration information. In the next step, the similarity matrix (Sim_MUT_) is generated using a novel feature selection approach based on the hidden Markov model and multinomial mixture model on single-nucleotide mutation data. Two similarity matrices (Sim_IC50_) and (Sim_TISSUE_) are achieved using IC50 values of cell lines across the drugs and tissue type information of each cell line, respectively. Finally, for constructing the latent vectors for each cell line and the drug and predicting whether the cell line is sensitive to the drug or not, the logistic matrix factorization method is applied. For assigning the IC50 values to two labels, sensitivity and resistance, we used the strategy introduced in previous researches [5, 7, 9], which used the median of the IC50 values for individual drugs as a threshold for the classification model. A cell line assigned to the sensitivity or class with label 1, if its IC50 value is smaller than the median of cell lines for an individual drug, and a cell line assigned to the resistance or class with label 0, otherwise. In the next section, we first describe the datasets used in the study and data preprocessing, and then the details of each above step are explained.

### Datasets and preprocessing

In this work, we use the GDSC dataset, consisting of 1001 cancer cell lines and 265 tested drugs and the CCLE dataset that has analyzed 1457 cancer cell lines and their genomic profiles against 24 drugs. In these datasets, cell lines were characterized by genomic features such as gene expression profile, copy number alteration, and single nucleotide mutation. The half-maximal inhibitory concentration (IC50) values are used for the sensitivity measure of cell lines across drugs. We focused on the 98 and 24 drugs for which SDF format (encoding the chemical structure of the drugs) were available from the NCBI PubChem Repository in GDSC and CCLE, respectively. There was no missing value in the gene expression features in these datasets. However, some cell lines have missing values for the response value, the single nucleotide mutation features, and the copy number alteration features. In the first step, the cell lines that contain missing values for more than half of the features were removed.

The known values of k-nearest neighbors imputed the remaining missing values. The Euclidean distance for each pair of cell lines c_i_ and c_j_ based on their gene expression profiles x_i_ and x_j_ are defined as follows:

1 $${\text{Dis}}_{{\text{E}}} \left( {{\text{c}}_{{\text{i}}} ,{\text{ c}}_{{\text{j}}} } \right) \, = \, \left| {\left| {{\text{ x}}_{{\text{i}}} {-}{\text{ x}}_{{\text{j}}} } \right|} \right|_{{2}}^{{2}}$$

Then the mean feature value among k-nearest cell lines for cell line c was used to impute the missing drug response value (IC50) of drug d as follows:

2 $${\text{IC5}}0\left( {{\text{c}},{\text{ d}}} \right) = \mathop \sum \limits_{{{\text{i}} = 1}}^{{\text{k}}} \frac{{Dis_{E} \left( {{\text{c}},{ }c_{i} } \right){ }}}{{\mathop \sum \nolimits_{{{\text{i}} = 1}}^{k} Dis_{E} \left( {{\text{c}},{ }c_{i} } \right){ }}}{\text{IC5}}0 \, \left( {{\text{c}}_{{\text{i}}} ,{\text{ d}}} \right)$$

Similarly, the mean feature value among k-nearest cell lines for cell line c was used to impute the missing copy number alteration value (CNV) of gene g as follows:

3 $${\text{CNV}}\left( {{\text{c}},{\text{ g}}} \right) = \mathop \sum \limits_{{{\text{i}} = 1}}^{{\text{k}}} \frac{{Dis_{E} \left( {{\text{c}},{ }c_{i} } \right){ }}}{{\mathop \sum \nolimits_{{{\text{i}} = 1}}^{k} Dis_{E} \left( {{\text{c}},{ }c_{i} } \right){ }}}{\text{CNV}} \, \left( {{\text{c}}_{{\text{i}}} ,{\text{ g}}} \right)$$

The values of single-nucleotide mutation features are binary-valued, i.e., 1 for mutation and 0 for wild type. The mean feature value among k-nearest cell lines for cell line c was considered to impute the missing MUT (single-nucleotide mutation) value of gene g as follows:

4 $${\text{MUT}}\left( {{\text{c}},{\text{ g}}} \right) = \left\{ {\begin{array}{*{20}l} 1 \hfill & {if\,\,\left( { \mathop \sum \limits_{{{\text{i}} = 1}}^{{\text{k}}} {\text{MUT}}\left( {c_{{\text{i }}} , {\text{g}}} \right) > \mathop \sum \limits_{{{\text{i}} = 1}}^{k} \left( {1 - {\text{MUT}}\left( {c_{{\text{i }}} , {\text{g}}} \right)} \right)} \right)} \hfill \\ 0 \hfill & {otherwise} \hfill \\ \end{array} } \right.$$

Finally, 555 cell lines and 98 drugs are considered for GDSC dataset and 363 cell lines and 24 drugs for CCLE dataset.

### Feature selection using autoencoder

Feature selection methods aim to reduce data dimensionality by identifying the subset of informative and non-redundant features in a dataset. Autoencoder is a non-recurrent neural network for unsupervised learning that reduces the datasets from initial feature space to a more significant feature space. It has an input layer, an output layer, and one or more hidden layers. The number of nodes (neurons) in the output layer is the same as in the input layer. Autoencoder learns the weight vector by assuming the output layer vector as the input layer vector. For constructing the autoencoder network for feature selection of the gene expression profile and copy number alteration information, the strategy introduced by Xu et al. [7] is used. Two autoencoder networks with a single hidden layer (with 100 neurons) and hyperbolic tangent as the activation functions are considered for screening out the gene expression features and copy number alteration data. After selecting the subset of features, a further small set of significant features was identified as two categories of inputs for the logistic matrix factorization model using the Boruta algorithm [6]. For determining the essential genes, the set of selected features by autoencoder networks along with the label of sensitivity and resistance corresponding to cell lines and drugs imported to the Boruta algorithm. Boruta algorithm is a wrapper built based on random forest classification that iteratively removes the less significant features by a statistical test. This algorithm added copies of all the features obtained using the autoencoder and it shuffled the values of the copied features for constructing shadow features, and it tried to find essential features. A random forest classifier is run on the extended information system, and Z-score values compute the importance of all attributes. Boruta algorithm repeats the finding procedure (finding the maximum Z-score among attributes) until the importance is assigned for all the attributes [6].

The first autoencoder with single-hidden-layer and Boruta algorithm are applied to the gene expression profile of 11, 712 and 19, 389 genes for two datasets GDSC and CCLE, respectively. The numbers of selected essential genes are 798 and 1189 for GDSC and CCLE, respectively. Also, the similar autoencoder and Boruta algorithm are applied to copy number alteration of 24, 959 and 24, 960 genes for two GDSC and CCLE datasets. 67 and 127 features selected for GDSC and CCLE datasets, respectively.

### Feature selection using hidden Markov model and multinomial mixture model

Understanding the activity of the mutational processes is critical for cancer treatment and personalized therapy. Since the mutational processes leave signatures of their activity in cancer genomes, characterizing the signatures of active mutational processes in patients from their patterns of single base substitutions is very important. In this study, we used the strategy proposed by Wojtowicz et al. for assigning the known signatures to the corresponding individual mutations for selecting essential mutated genes in cancer types [10]. In this work, we consider only the validated mutation signatures of the Catalogue of Somatic Mutations in Cancer (COSMIC) [11], and we focused on the signatures previously identified as active in cancer types [12]. Table 1 shows the active signatures of 14 cancer types corresponding to cancer cell lines in GDSC and CCLE datasets (only the cancer types with at least 15 cell lines in GDSC and CCLE datasets are considered).

**Table 1:** The active signatures of 14 cancer types corresponding to cell lines in GDSC and CCLE datasets

BRCA	BLCA	ESCA	HNSC	LUAD	OV	SKCM	STAD	COAD	PACA	MALY	LIHC	BONE	CESC
1	1	1	1	1	1	1	1	1	1	1	1	1	1
2	2	2	2	2	2	2	2	2	2	2	4	5	2
3	5	4	4	4	3	5	3	5	3	5	5	6	5
5	10	5	5	5	6	7	5	6	5	9	12	7	13
6	13	6	7	6	13	11	6	7	6	13	16	13	26
8	22	7	13	15	15	13	10	10	13	17	17	30	–
13	–	13	15	17	18	17	13	14	–	–	22	–	–
17	–	15	16	18	21	–	15	15	–	–	23	–	–
18	–	16	18	21	26	–	17	21	–	–	–	–	–
20	–	17	21	–	–	–	21	26	–	–	–	–	–
26	–	21	26	–	–	–	26	28	–	–	–	–	–
30	–	26	–	–	–	–	–	30	–	–	–	–	–

Because there are six classes of base substitution (C:G > A:T, C:G > T:A, C:G > G:C, A:T > C:G, A:T > T:A, A:T > G:C) and four possible 5′, we categorized mutations in a cancer genome into 96 categories that include its base substitution and four possible 3′ bases [13, 14]. We downloaded single base substitutions of cancer types from the International Cancer Genome Consortium Data Portal [15]. We analyzed single base substitutions of several patients from considered cancer types, and the number of these patients (patient group1) corresponding to each cancer type is shown in Table 2. For each cancer type, the following hidden Markov model and multinomial mixture model are applied, and the important genes for the considered cancer type will be determined. In this model, the number of states for each cancer type is determined based on the number of the corresponding signature that is shown in Table 1. For example, the number of states (t) in BRCA cancer is 12.

**Table 2:** The number of patients for learning the HMM and MMM models (patient group1) and the number of patients whose gene expression information is available in the International Cancer Genome Consortium Data Portal (patient group2) for 14 cancer types

Cancer type	Patient group1	Patient group2
BRCA	560	560
BLCA	320	240
ESCA	190	150
HNSC	180	125
LUAD	280	197
OV	200	170
SKCM	135	135
STAD	245	120
COAD	140	127
PACA	160	160
MALY	241	241
LIHC	250	210
BONE	280	225
CESC	223	190

The detailed step-wise feature selection procedure is described as follows:

#### Identifying close and isolated mutations

We classified the mutations into two classes, close and isolated mutations, using a distance threshold of 2000 bp (isolated mutations are distant from any other mutation). We set the first mutation of each mutation sequence to close. For other mutations, if the corresponding distance to the previous mutation is greater than 2000 bp, the mutation is labeled as isolated, and close otherwise. Therefore from a sequence of mutation of the patient, we can obtain several subsequences, some corresponding to close and some corresponding to isolated mutations. For example, two subsequences corresponding to the isolated, and three subsequences corresponding to the close mutations of a patient with BRCA cancer is as follows:

5 $$\begin{aligned} & \underbrace {T > G,T > C,G > A,C > G,T > C,}_{close}\underbrace {G > T,T > C,,C > G, \ldots ,}_{isolated} \\ & ,\underbrace {C > G,T > C,C > G,C > T,G > A,}_{close}\underbrace {G > T,C > G,C > G,}_{isolated}\underbrace {T > C.}_{close} \\ \end{aligned}$$

#### Modeling close mutations

Since the subsequences corresponding to close mutations are close to each other, it can be assumed that there is a dependency between them. So these subsequences were modeled using a hidden Markov model (HMM).

An HMM M with t (the number of mutation signatures) hidden states is represented by

- Σ = {c_1_, …, c_s_} is the set of alphabets of all sequences.
- Q = {q_1_, …, q_t_} is the set of states, each of which is able to emit symbols of alphabet Σ.
- π_i_, ∀i = 1, …, t is the probability to start with ith state.
- A = [a_i,j_]_i,j=1,….,t_ which a_i,j_ is the transition probability from q_i_ to q_j_.
- E = [e_i,j_]_i=1,…,t, j=1,…,s_ where e_i,j_ is the probability that state q_i_ emits c_j_.

The model assumes that each observation, representing a mutation category, is emitted by one of the t states. The sequence of states that generated the observed sequence is unknown, and each state depends on the previous state. For learning the parameters of the model, π, A, E, all obtained close subsequences in the first step considered as the training set for estimation of the parameters of HMM. In this study, the AntMarkov algorithm (the algorithm for parameter estimation of Hidden Markov Model inspired by Ant Colony Optimization) [16] was applied to estimate HMM parameters.

#### Modeling isolated mutations

Since the isolated subsequences are distant from any other mutation, the assumption of dependency between them is less motivated. So, isolated mutations were modeled using a multinomial mixture model (MMM). An MMM is parameterized by a vector g of t mutation signature marginal probabilities and a t × s emission matrix E, (s = 96). All obtained isolated subsequences in the first step considered as the training set for estimation of the parameters of MMM. The vector g and emission matrix E were estimated based on counting the number of times the isolated mutations were observed in samples (experimental distribution). We consider a vector T with size 96, which T[i] is the total number of times the ith mutation category were observed as isolated mutations in samples. By applying vector T to the initialized vector g and emission matrix E, their estimated values were achieved.

#### Computing mutation sequence occurrence

After training two above models, the probability of sequence occurrence O_1_, …, O_T_, which is decomposed into close and isolated subsequences,{C_1_, I_1_, C_2_, I_2_, …, C_k1_, I_k2_}, is formulated as follows:

6 $${\text{P}} = \left( {\mathop \prod \limits_{{{\text{i}} = 1}}^{{{\text{k}}1}} P{\text{(C}}_{{\text{i }}} {\text{|HMM}})} \right){*}\left( {{ }\mathop \prod \limits_{{{\text{j}} = 1}}^{{{\text{k}}2}} P{\text{(I}}_{{\text{j }}} {\text{|MMM}})} \right){ }$$

The Viterbi algorithm [17] was applied to find the paths of the most likely sequence of states that generated the close subsequences. To determine the most probable paths corresponding to isolated mutations, the estimated values of g vector and emission matrix, E are used. As for each isolated mutation category (O_t_), the state with maximum probability value (Q_t_) is obtained from the following formula:

7 $${\text{max}}_{j = 1 \ldots .t} \left( {{\text{P}}\left( {{\text{Q}}_{{\text{j }}} } \right)*{\text{P }}\left( {{\text{O}}_{{\text{j }}} {\text{| Q}}_{{\text{j }}} } \right)} \right)$$

Finally, we append these two most probable paths of states to construct the final path corresponding to the patient. Then, the numbers of observed states (signature) are calculated as signature frequency per sample or signature activities for each path. For example, a patient with BRCA cancer has a vector with size 12 corresponding to the number of signatures, and the elements of this vector are calculated based on the number of times each state is observed in the final path.

#### Identifying the important genes

For considering the relationship between signature activities and gene expression profiles for each patient, we downloaded the gene expression files for patients from the International Cancer Genome Consortium Data Portal [15]. The number of patients whose gene expression information is available (patient group2) is shown in Table 2. Also, since the information of single nucleotide mutation of 54 and 1667 genes for two GDSC and CCLE datasets were accessible, we analyzed these genes’ expression for computing the Spearman correlation coefficients. Therefore, the Spearman correlation coefficients between the expression of 1721 genes and signature activities across samples are calculated. In this way, we identified essential genes with a high Spearman correlation coefficient (greater than 0.2) in close and isolated regions in each cancer type. The results of the correlation between some genes and signature activity in 14 cancer types that have high Spearman correlation coefficients are illustrated in the Additional file 1: Table-S1. We considered these genes as essential features for single nucleotide mutation data in GDSC and CCLE datasets. Finally, 22 and 72 genes are selected by the above strategy based on a hidden Markov model and multinomial mixture model in GDSC and CCLE, respectively. The list of these genes is illustrated in the Additional file 1: Table S1.

### Similarity definition

Since similar cell lines and similar drugs may have similar drug responses, the similarities between cell lines and drugs can improve drug response prediction [5, 18].

The similarity matrices are required for the identification of the nearest neighbors in the logistic matrix factorization model. Gene expression profile, copy number alteration, single-nucleotide mutation, and tissue type information are used for cell line similarity, and chemical structures of drugs are used for drug similarity. So, five similarities between each pair of cell lines and the similarity between each pair of drugs are defined as follows:

#### Cell line similarity

- (Sim_EXP_) is the similarity based on the selected features of the gene expression profile, in which the numbers of identified essential genes for gene expression profile by autoencoder are 798 and 1189 for two datasets GDSC and CCLE, respectively. Sim_EXP_ is defined as the Pearson correlation between the gene expression vector of each pair of n cell lines, arranged in an n × n matrix.
- (Sim_CNV_) is the similarity based on the selected features of copy number alteration data, which 67 and 127 useful features selected by autoencoder in GDSC and CCLE, respectively. Sim_CNV_ matrix is defined as an n × n matrix by Pearson correlation between the copy number alteration vector of each pair of cell lines.
- (Sim_MUT_) is the similarity based on the selected features of single nucleotide mutation information by the hidden Markov model and multinomial mixture model. 22 and 67 essential genes identified by this strategy from GDSC and CCLE datasets, respectively. Then, the Jaccard similarity is applied on each pair of single nucleotide mutation vectors corresponding to n cell lines, and Sim_MUT_ is constructed as an n × n matrix.
- (Sim_IC50_) is the similarity between cell lines based on their IC50 values. This definition of similarity between cell lines proposed by Liu is based on the correlation between response IC50 values of the cell lines [19]. Sim_IC50_ is defined as the Pearson correlation between each of the n cell lines considered an n × n matrix.
- (Sim_TISSUE_) is the similarity between cell lines based on tissue type. The complete set of samples consisted of GDSC and CCLE datasets cancer cell lines originated from around 14 tissue sites. Sim_TISSUE_ is an n × n binary-valued matrix, which for entry corresponding to row i and column j is 1, if two cell lines c_i_ and c_j_ have the same tissue type and zero otherwise. The Sim_TISSUE_ matrices corresponding to GDSC and CCLE cell lines are represented in the Additional file 2: Table-S2 and Additional file 3: Table-S3.

Since the correlation coefficient between each pair of the above similarity matrices is very low, there is no collinearity between matrices, and they can be linearly combined. We constructed an integrated matrix similarity, Sim_CL_ = [SC_ij_]_n×n_, using the combination of Sim_EXP_, Sim_CNV_, Sim_MUT_, Sim_IC50_ and Sim_TISSUE_ by the following formula:

8 $$\frac{{\uplambda {\text{Sim}}_{{{\text{EXP}} }} +\upgamma {\text{Sim}}_{{{\text{CNV}} }} +\upphi {\text{Sim}}_{{{\text{MUT}} }} +\uppsi {\text{Sim}}_{{{\text{IC}}50 }} + \uprho {\text{Sim}}_{{{\text{TISSUE}}}} }}{{\uplambda + \upgamma + \upphi + \uppsi + \uprho }}$$

where γ, λ, ϕ, ψ, and ρ are parameters that control the importance of each of the matrix and tuned in the model. We defined the set N_k_(c_i_) that denotes the k-most similar cell lines to c_i_ (except c_i_) using (Sim_CL_) matrix. We constructed adjacency matrix A = [a_ij_]_n×n_ that represents cell line neighborhood information as follow:

9 $${\text{a}}_{{{\text{ij}}}} = \left\{ {\begin{array}{*{20}l} {SC_{{{\text{ij}}}} } \hfill & {c_{{\text{j }}} \in {\text{N}}_{{\text{k }}} \left( {c_{{\text{i }}} } \right)} \hfill \\ 0 \hfill & {otherwise} \hfill \\ \end{array} } \right.$$

#### Drug similarity

The similarity between drugs is constructed based on chemical substructures (Sim_DRUG_). For each drug, a zero–one vector of size 881 is considered where 881 is the number of known chemical substructures of a drug. In this vector, 1 indicates the presence of a substructure of the drug and 0 otherwise. Sim_DRUG_ = [SD_ij_]_m×m_ is constructed as an m × m matrix by Jaccard similarity between each of the chemical substructures vector corresponding to the m drugs. For a drug d_i_, the set N_k_(d_i_) denotes the k-most similar drugs to d_i_ (except d_i_) using Sim_DRUG_ matrix. The adjacency matrix, B = [b_ij_]_m×m_, describes the drug neighborhood information as follows:

10 $${\text{b}}_{{{\text{ij}}}} = \left\{ {\begin{array}{*{20}l} {SD_{{{\text{ij}}}} } \hfill & {d_{{\text{j }}} \in {\text{N}}_{{\text{k }}} \left( {d_{{\text{i }}} } \right) } \hfill \\ 0 \hfill & {otherwise } \hfill \\ \end{array} } \right.$$

### Logistic matrix factorization

For drug response prediction of cancer cell lines from GDSC and CCLE datasets using selected features, the DSPLMF method introduced based on the logistic matrix factorization method [5] is applied based on the following objective function:

11 $$\begin{aligned} & {\text{min}}_{{{\text{U}},{\text{V}},\upbeta ^{c} , \upbeta ^{d} }} \mathop \sum \limits_{{{\text{i}} = 1}}^{n} \mathop \sum \limits_{{{\text{j}} = 1}}^{m} \left( {1 + rq_{{\text{ij }}} - { }q_{{\text{ij }}} } \right)\log \left( {1 + \exp \left( {{\text{u}}_{{\text{i }}} {\text{v}}_{{\text{j }}}^{T} + { }\upbeta _{{\text{i }}}^{c} +\upbeta _{{\text{j }}}^{d} { }} \right)} \right) \\ & \quad - \,rq_{{\text{ij }}} \left( {{\text{u}}_{{\text{i }}} {\text{v}}_{{\text{j }}}^{T} +\upbeta _{{\text{i }}}^{c} +\upbeta _{{\text{j }}}^{d} } \right) + \frac{1}{2} tr[ {\text{U}}^{T} (\uplambda _{{\text{c }}} I + \upalpha {\text{H}}^{c} ){\text{ U}}] + { }\frac{1}{2} tr [{\text{V}}^{T} (\uplambda _{{\text{d }}} I +\upbeta {\text{H}}^{d} ){\text{ V }}] \\ \end{aligned}$$

where u_i_ and v_j_ are the latent vectors of size L corresponding to the cell line c_i_ and drug d_j_, respectively and the latent vectors of all cell lines and all drugs are denoted by U and V. The positive values ${\upbeta }_{{\text{i }}}^{c}$ and ${\upbeta }_{{\text{j }}}^{d}$ are the bias parameters according to cell line c_i_ and drug d_j_ and ${\upbeta }_{ }^{c} {\text{and}} {\upbeta }_{ }^{d}$ are the bias vectors for cell lines and drugs, respectively [20]. Two parameters, λ_c_ = $\frac{1}{{{\upsigma }_{{\text{c }}}^{2} }}$, λ_d_ = $\frac{1}{{{\upsigma }_{{\text{d }}}^{2} }}$, where ${\upsigma }_{{\text{c }}}^{2}$ and ${\upsigma }_{{\text{d }}}^{2}$ are parameters for controlling the variances of prior distributions of cell lines and drugs. The parameters α and β determine the effectiveness of cell line similarity and drug similarity in the DSPLMF method. (r ≥ 1) is a parameter for controlling the importance levels of observed interactions. Since both sensitivity and resistance classes have the same importance in drug response prediction problem, we set r to be one. Also, H^c^ = (E^c^ + $\widetilde{{{\text{E}}^{c} }}$) − (A + A^T^), E^c^ and $\widetilde{{{\text{E}}^{c} }}$ are two diagonal matrices with ${\text{E}}_{{\text{ii }}}^{c} = \sum\nolimits_{{{\text{j}} = 1}}^{{\text{n}}} {({\text{a}}_{{\text{ij }}} )}$ and $\widetilde{{{\text{E}}_{{\text{ jj }}}^{c} }} = \sum\nolimits_{{{\text{i}} = 1}}^{{\text{n}}} {({\text{a}}_{{\text{ij }}} )}$, H^d^ = (E^d^ + $\widetilde{{{\text{E}}^{d} }}$) − (B + B^T^) as diagonal elements (n is the numbers of cell lines). E^d^ and $\widetilde{{{\text{E}}^{d} }}$ are two diagonal matrices with ${\text{E}}_{{\text{ii }}}^{d} = \sum\nolimits_{{{\text{j}} = 1}}^{{\text{m}}} {({\text{b}}_{{\text{ij }}} )}$ and $\widetilde{{{\text{E}}_{{\text{ jj }}}^{d} }} = \sum\nolimits_{{{\text{i}} = 1}}^{{\text{m}}} {({\text{b}}_{{\text{ij }}} )}$, as diagonal elements (m is the numbers of drugs). After training the proposed model, the latent vectors of cell lines and drugs are determined using the formula 11. Then, for predicting the IC50 values of a given new cell line across all drugs, the k-nearest neighbors for the new cell line are selected, and the latent vector for this new cell line is estimated based on the average of latent vectors of its neighbors. Since the elements of (Sim_IC50_) matrix are unknown, the (Sim_CL_) matrix cannot be used for finding the k-nearest neighbors for the new cell line. We used the strategy introduced in the DSPLMF method for estimation (Sim_IC50_) matrix. DSPLMF method is designed a Decision Tree Classifier model for estimation (Sim_IC50_) matrix using the gene expression profile, copy number alteration, and single-nucleotide mutation information of the new cell line [5]. Then by a similar method, we estimated the latent vector corresponding to the new cell line to predict the probabilities that the new cell line is sensitive to drugs indicated by Eq. 12. For the set of cell lines and drugs, the probability of the cell line c_i_ is sensitive to the drug d_j_ can be modeled as a logistic function as follows:

12 $${\text{p}}_{{{\text{ij}}}} = \frac{{\exp \left( {{\text{u}}_{{\text{i }}} {\text{v}}_{{\text{j }}}^{T} + { }\upbeta _{{\text{i }}}^{c} +\upbeta _{{\text{j }}}^{d} } \right)}}{{1 + {\text{exp}}\left( {{\text{u}}_{{\text{i }}} {\text{v}}_{{\text{j }}}^{T} +\upbeta _{{\text{i }}}^{c} +\upbeta _{{\text{j }}}^{d} } \right)}}$$

Finally, a threshold is applied on probabilities to assign a sensitive or resistance class to each new cell line-drug pair.

## Results

### Evaluation of prediction performance of Auto-HMM-LMF

Using the feature selection approaches is one of the common methods to reduce the dimensions of the features in drug response prediction problems. In some of the previous predictive methods, such as AutoBorutaRF, the autoencoder approaches are used for selecting significant features of genomic information. One of the most powerful methods of selecting features is the EFS method proposed by Neumann et al. The EFS method integrated eight different feature selection methods and normalized all individual outputs to a common scale, an interval from 0 to 1 [21, 22]. First, to evaluate the efficiency of the feature selection strategies in the Auto-HMM-LMF model, we use the EFS method to select important features in the gene expression profile, copy number variation and single nucleotide mutation data. In this method, the number of features selected for each group of data is equal to the number of features selected by the Auto-HMM-LMF method. Then we alternated these features with features selected by Autoencoder and HMM-MMM in the Auto-HMM-LMF method, and we compared the achieved results to other methods. This method is applied to two CCLE and GDSC datasets, and we represent the results of this approach by the name of EFS-LMF in Tables 3 and 4. In this study, the tenfold cross-validation is repeated 30 times, and the mean value of them is used as criteria for evaluating the predictive performance of the AutoHMM-LMF method.

**Table 3:** Performance comparison of the different algorithms results based on seven metrics on GDSC dataset

Method	Accuracy	Recall	Precision	Specificity	F1Score	MCC	AUC
Auto-HMM-LMF	**0.70**	**0.78**	**0.68**	0.63	**0.73**	**0.39**	**0.78**
DSPLMF	0.68	0.75	0.67	0.61	0.70	0.37	0.76
EFS-LMF	0.67	0.72	0.67	0.64	0.68	0.35	0.77
CaDRReS	0.54	0.54	0.54	0.54	0.55	0.12	0.51
SRMF	0.51	0.52	0.52	0.51	0.51	0.10	0.49
AutoBorutaRF	0.65	0.65	0.64	**0.65**	0.65	0.31	0.71
SVM-RFE	0.59	0.58	0.58	0.61	0.58	0.19	0.51

**Table 4:** Performance comparison of the different algorithms results based on seven metrics on CCLE dataset

Method	Accuracy	Recall	Precision	Specificity	F1Score	MCC	AUC
Auto-HMM-LMF	**0.79**	**0.72**	**0.69**	**0.84**	**0.70**	**0.53**	**0.83**
DSPLMF	0.77	0.72	0.63	0.77	0.67	0.48	0.77
EFS-LMF	0.76	0.67	0.66	0.82	0.65	0.47	0.78
CaDRReS	0.67	0.35	0.49	0.83	0.41	0.20	0.50
SRMF	0.51	0.45	0.34	0.52	0.41	0.10	0.49
AutoBorutaRF	0.76	0.65	0.59	0.81	0.62	0.45	0.82
SVM-RFE	0.73	0.43	0.63	0.81	0.52	0.29	0.55

We compared the Auto-HMM-LMF method to six classification models, DSPLMF, EFS-LMF, CaDRReS, SRMF, AutoBorutaRF, and SVM-RFE for different metrics. DSPLMF and AutoBorutaRF are designed as the classification models, but the CaDRReS and SRMF methods predicted IC50 values as output. So, for comparison of these models with the Auto-HMM-LMF and EFS-LMF methods, we applied the median of predicted IC50 values for each drug as a classification threshold. If the predicted IC50 value corresponding to a cell line-drug pair is smaller than this threshold, the sensitive class was assigned to it; otherwise, it was labeled with resistance class. Seven metrics Accuracy, Recall, Precision, Specificity, F1Score, Matthews correlation coefficient (MCC) and area under the receiver operating characteristic curve (AUC) are used that; these criteria are formulated as follows:

$${\text{Accuracy}} = \frac{{{\text{T P }} + {\text{ T N }}}}{{{\text{T P }} + {\text{ F P }} + {\text{ T N }} + {\text{ F N}}}}$$

$${\text{Recall}} = \frac{{\text{T P}}}{{{\text{T P }} + {\text{ F N}}}}$$

$${\text{Precision}} = \frac{{\text{T P}}}{{{\text{T P }} + {\text{ F P}}}}$$

$${\text{Specificity}} = \frac{{\text{T N}}}{{{\text{T N}} + {\text{ F P}}}}$$

$${\text{F1Score}} = \frac{{2{\text{T P}}}}{{2{\text{T P }} + {\text{ FP}} + {\text{F N}}}}$$

13 $${\text{MCC}} = { }\frac{{{\text{T P * T N }} - {\text{ F P * F N }}}}{{\sqrt {\left( {{\text{TP}} + {\text{FP}}} \right)\left( {{\text{TP}} + {\text{FN}}} \right)\left( {{\text{FP}} + {\text{TN}}} \right)\left( {{\text{FN}} + {\text{TN}}} \right)} }}$$

where

- TP (true positive): The number of cell lines labeled with sensitivity and predicted as sensitivity.
- TN (true negative): The number of cell lines labeled with resistance and predicted as resistance.
- FP (false positive): The number of cell lines labeled with resistance and predicted as sensitivity.
- FN (false negative): The number of cell lines labeled with sensitivity and predicted as resistance.

Tables 3 and 4 show the results of comparative experiments conducted on the GDSC and CCLE datasets (the bold number represents the best result). As is shown in Table 3, the value of Accuracy, Recall, Precision, F1Score, MCC, and AUC criteria have increased by 0.02, 0.03, 0.01, 0.03, 0.02, and 0.02 compared to the best algorithm, DSPLMF. In the Specificity criterion, the AutoBorutaRF method performs significantly better than the other methods. Concerning the other criteria, the Auto-HMM-LMF method has very significant results to the results of the AutoBorutaRF method. In Table 4, the value of all criteria by Auto-HMM-LMF has increased compared to the result of other algorithms, and Auto-HMM-LMF significantly outperformed the state-of-the-art-methods in this dataset. As is shown in Tables 3 and 4, the value of all criteria by Auto-HMM-LMF has increased compared to the result of other algorithms. These observations demonstrated that the selected features by HMM and MMM strategies for mutation data and autoencoder technique for gene expression and copy number variation data are very effective and essential. Also, the features selected by the EFS method cannot be as powerful as the features selected by the Auto-HMM-LMF method in predicting drug response.

### Tissue specific of cell line type

To demonstrate the Auto-HMM-LMF method’s performance in different tissue types, we examine whether our proposed method can achieve good performance when considering specific cell line tissue types. In this way, 73 Haematopoietic and lymphoid cell lines in the GDSC dataset are considered, and seven criteria evaluate the Auto-HMM-LMF method. We trained the Auto-HMM-LMF method by these cell lines, and we applied a tenfold cross-validation approach for drug response prediction of considered cell lines. As is shown in Table 5, these results justify that the Auto-HMM-LMF method can also achieve consistently or, in some criteria, more performance on Haematopoietic and lymphoid cell lines.

**Table 5:** Prediction performance of Auto-HMM-LMF method on 73 Haematopoietic cell lines from GDSC dataset based on seven criteria

Method	Accuracy	Recall	Precision	Specificity	F1Score	MCC	AUC
Auto-HMM-LMF	0.71	0.81	0.69	0.63	0.74	0.44	0.78

### Correlation between predicted and observed responses values

**Fig. 2:**
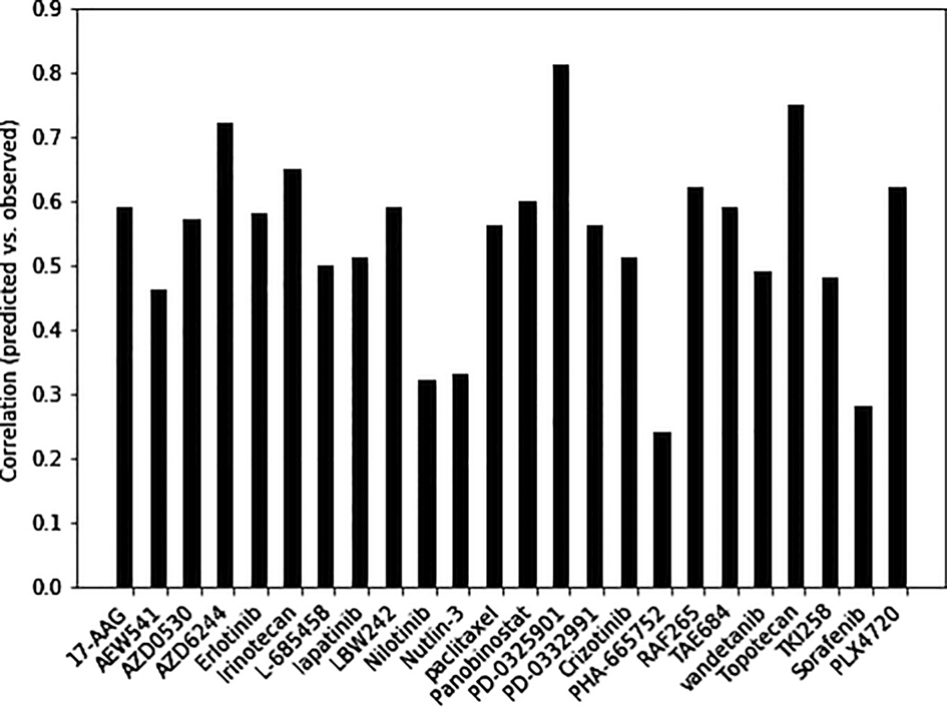
Pearson correlation coefficients between predicted and observed response values for 24 drugs in CCLE using Auto-HMM-LMF model

**Fig. 3:**
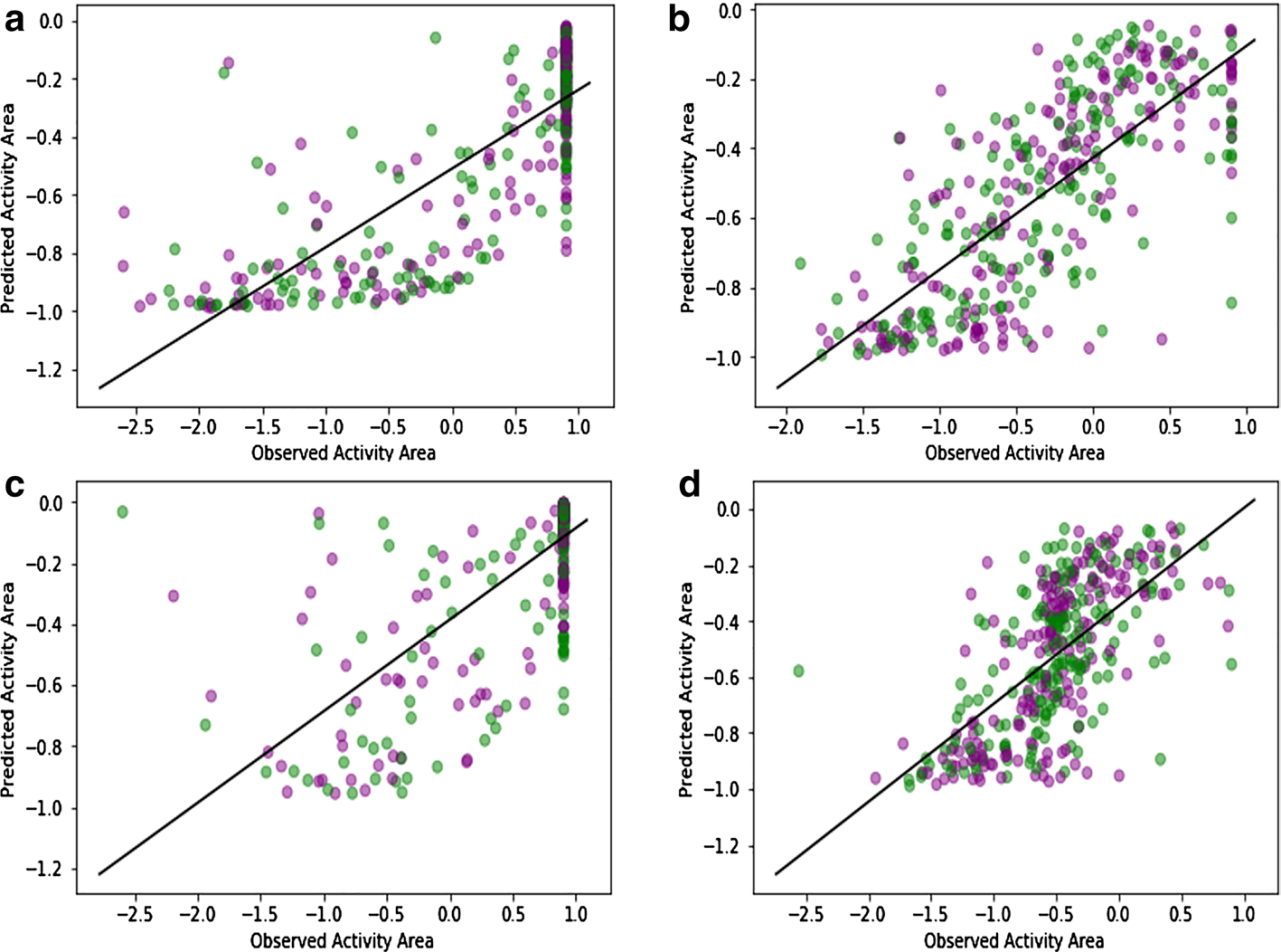
Correlations between observed and predicted activity areas for CCLE cell lines across 4 drugs using Auto-HMM-LMF method. The green points represent the observed values and purple points represent the predicted values by Auto-HMM-LMF method. **a** The scatter plot of observed and predicted drug responses for PD—0325901 (Pearson correlation coefficient = 0.81). **b** The scatter plot of observed and predicted drug responses for Topotecan (Pearson correlation coefficient = 0.75). **c** The scatter plot of observed and predicted drug responses for AZD6244 (Pearson correlation coefficient = 0.72). **d** The scatter plot of observed and predicted drug responses for Irinotecan (Pearson correlation coefficient = 0.65)

We plotted the Bar chart of Pearson correlation coefficients of observed drug responses and predicted values for 24 drugs in the CCLE dataset. As is shown in Fig. 2, (70%) above correlation coefficients, 17 from 24, are higher than 0.5. For four of these drugs (PD—0325901, T opotecan, AZD6244 and Irinotecan) correlation coefficients are greater than 0.65. These plots show the excellent performance of the Auto-HMM-LMF method in predicting drug response values. The scatter plots of observed and predicted drug responses by the Auto-HMM-LMF model of 4 above drugs are drawn in Fig. 3, and the scatter plots of 20 other drugs in the CCLE dataset are illustrated in the Additional file 4.

### Application for drug repositioning

**Fig. 4:**
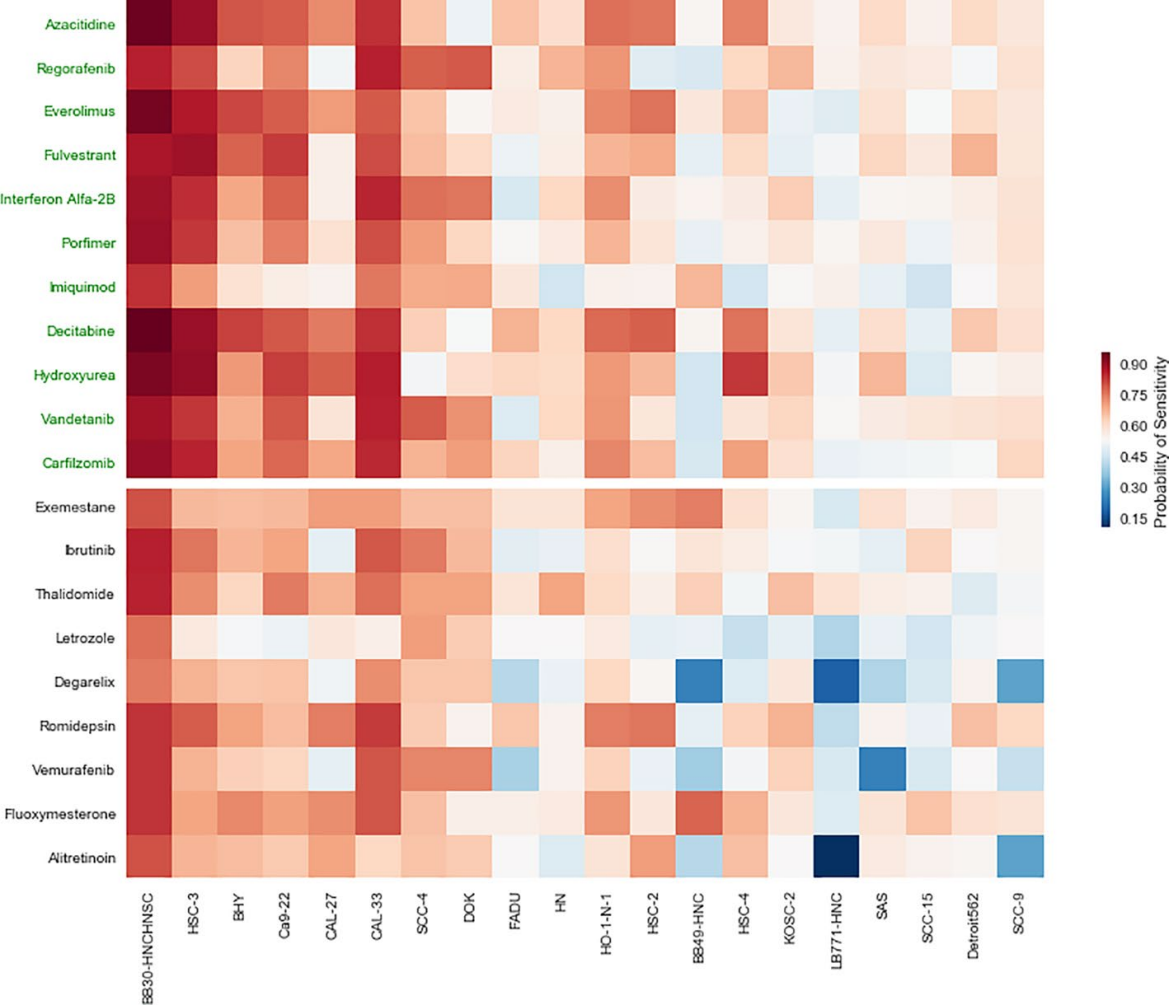
The probability of sensitivity to 20 anticancer drugs of 20 HNSC cell lines. Drugs whose names are shown in green have been studied for the treatment of HNSC. The probability higher than 0.5 means that the corresponding row drug may be a novel repositioned drug for the treatment of the corresponding column cell line. Using a diverging color scheme, all values around 0.5 are ignored, and visual importance on either highly probable in red versus low probability in blue are added

Drug repositioning is the process of selecting a known drug for an alternative pharmacological purpose. For this issue, we considered 37 US Food and Drug Administration (FDA) approved drugs that were not tested in the GDSC dataset from the study of Choi et al. [23]. The Auto-HMM-LMF model was trained on the GDSC dataset and the probability of sensitivity of 20 cell lines of head and neck cancer (HNSC) across 20 anticancer drugs of 37 drugs were predicted and were shown in Fig. 4.

As it can be seen in Fig. 4, 11 following drugs have been identified as an effective treatment of HNSC:

- Azacitidine: Azacitidine is a type of drug called a hypomethylating agent, and a study reported that Azacitidine and Cisplatin are effective for the treatment of head and neck cancer [24].
- Regorafenib: Regorafenib is an oral multi-kinase inhibitor that targets receptor tyrosine kinase (RTK). Klinghammer et al. established a panel of 65 head and neck squamous cell carcinoma, and they demonstrated that combinational treatment of regorafenib and Everolimus is useful in these patients [25].
- Everolimus: Everolimus is used as an immunosuppressant to prevent rejection of organ transplants in the treatment of cancer. Recently, a study showed that patients with TP53 mutations benefited significantly from Everolimus in head and neck cancer [26].
- Fulvestrant: Fulvestrant is a drug used to treat hormone receptor (HR)-positive metastatic. Grünow et al. [27] showed that Fulvestrant inhibits irradiationinduced ESR2 expression, and their findings demonstrated the efficacy of Fulvestrant in combination with radiotherapy for HNSC patients.
- InterferonAlf a–2B: InterferonAlf a–2B is an antiviral or antineoplastic drug that is an effective treatment in head and neck cancer [28].
- Porfimer: Porfimer is a photosensitizer, and it is used in radiation therapy in cancer treatment. An in vivo study suggested that this drug can be used in treatment for HNSC patients [29].
- lmiquimod: Imiquimod (INN) is a prescription drug that acts as an immune response modifier used to treat basal cell carcinoma. The study showed that topical Imiquimod might offer a reasonable and well-tolerated palliative treatment option for patients [30].
- Decitabine or 5–aza–2′–deoxycytidine is a nucleic acid synthesis inhibitor for cancer treatment. Cisplatin resistance in head and neck squamous cell carcinoma reduces survival. Viet et al. [31] showed that Decitabine treatment restored Cisplatin sensitivity in HNSC cell lines and significantly reduced the Cisplatin dose required to induce apoptosis.
- Hydroxyurea: Hydroxyurea is an anti-cancer agent used to treat melanoma, resistant, recurrent, and metastatic cancer types. A study displayed Hydroxyurea is a single active agent in head and neck cancer. It has been used clinically as a radiation-enhancing drug with radiotherapy [32].
- V andetanib: V andetanib acts as a kinase inhibitor of several cell receptors, and it is an anti-cancer drug for the treatment of cancer cell lines. Sano et al. [33] approved the addition of V andetanib to combination therapy with Cisplatin, and radiation can overcome resistance in vitro and in vivo models of HNSC.
- Carf ilzomib: Carf ilzomib is an anti-cancer drug acting as a selective proteasome inhibitor. By upregulation of pro-apoptotic Bik, Carf ilzomib and ONX0912 potently induced apoptosis in HNSC cell lines [34].

These results indicate that the Auto-HMM-LMF model can be useful in drug repositioning. Also, five drugs (Exemestane, Ibrutinib, T halidomide, Romidepsin and Fluoxymesterone) may be novel therapeutic drugs for HNSC.

### Hyperparameters settings

Since the numbers of cell lines and drugs in the GDSC dataset are higher than the CCLE dataset, we tuned the hyperparameters on the GDSC dataset, and we used the obtained values of the hyperparameters in both datasets. In this way, the tenfold cross-validation procedure is applied to GDSC, and hyperparameters are determined by maximizing the AUC criterion.

The learned hyperparameters using the GDSC dataset are shown in Table 6. The threshold parameter applied on Eq. 12 for determining the label of the class for each new cell line was chosen from {0.1, …,1} and this parameter was set to 0.4. The latent space dimension L was chosen from {1, …, min(n, m)}, for GDSC dataset L parameter was set to 95 and for CCLE dataset L was set to 23 (where n and m are the numbers of cell lines and the numbers of drugs, respectively).

**Table 6:** Learned hyperparameters of Auto-HMM-LMF method based on GDSC dataset

Hyperparameter	Description	Value
k	Number of nearest neighbors (Eq. 9)	20
α	Effectiveness of cell line similarity (Eq. 11)	0.5
β	Effectiveness of drug similarity (Eq. 11)	0.1
λ_c_	Variance parameter of cell lines (Eq. 11)	0.5
λ_d_	Variance parameter of drugs (Eq. 11)	0.5
λ	Importance of Sim_EXP_ (Eq. 8)	2
γ	Importance of Sim_CNV_ (Eq. 8)	2
ϕ	Importance of Sim_MUT_ (Eq. 8)	2
ψ	Importance of Sim_IC50_ (Eq. 8)	5
ρ	Importance of Sim_TISSUE_ (Eq. 8)	2
threshold	Threshold parameter	0.4

The parameter k were selected from 1 to 50. The impact factors of nearest neighbors α and β in equations were selected from {2^–5^, 2^–4^, …, 2^2^}. The variance parameters, λ_c_ and λ_d_, were chosen from {2^–5^, 2^–4^, …, 2^1^}. The five parameters γ, λ, ϕ, ψ, and ρ were selected from 1 to 5

## Discussion

**Fig. 5:**
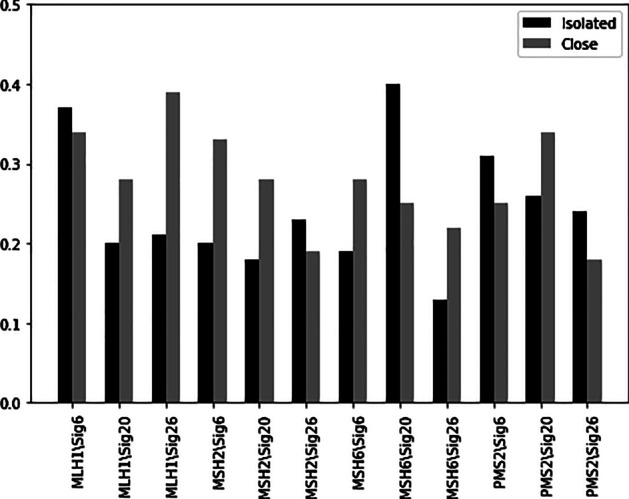
Spearman correlation comparison of the PMS2, MLH1, MSH2, and MSH6 expression with signature 6, 20, and 26 activities across BRCA samples. For these three signatures, the mutation counts in both isolated and close are positively correlated with expression of considered genes

This paper proposed the Auto-HMM-LMF method based on feature selection approaches and logistic matrix factorization strategy to predict drug response. The proposed prediction model showed higher predictive efficiency than the existing computational models. Also, we demonstrated that the Auto-HMM-LMF model could be useful in drug repositioning. So, we identified five drugs (Exemestane, Ibrutinib, T halidomide, Romidepsin and Fluoxymesterone) for HNSC treatment. To illustrate the biological significance of the features selected by the hidden Markov model (HMM) and multinomial mixture model (MMM) on mutation data in the Auto-HMMLMF method, we further consider cancer cell lines related to breast cancer (BRCA) and two important processes, namely MMR and HRD. This study selected 30 significant genes by considering the Spearman correlation coefficient between their gene expression file and signature activity for 12 signatures of BRCA cancer cell lines. Among these genes, the gene expression of four genes namely PMS2, MLH1, MSH2, and MSH6 has high Spearman correlation coefficient with signatures 6, 20, and 26 activities. The results of the Spearman correlation between the expression of these genes and three mutation signature activities in BRCA are shown in Fig. 5. On the other hand, a recent study [35] has shown that the three 6, 20, and 26 signatures are associated with MMR deficiency in breast cancer. Defective DNA mismatch repair (MMR) occurs in many cancer types, and mutations in the PMS2, MLH1, MSH2, and MSH6 genes are the most common cause of mismatch repair (MMR) deficient. The above genes are known as DNA mismatch repair (MMR) genes, and these genes are involved in repairing errors in DNA replication (the errors that occur when DNA is copied in preparation for cell division) [36].

**Fig. 6:**
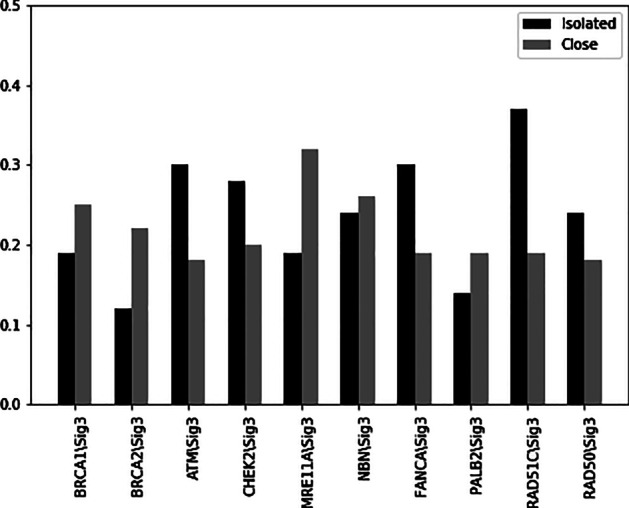
Spearman correlation comparison of the BRCA1, BRCA2, ATM, CHEK2, MRE11A, NBN, FANCA, PALB2, RAD51C, RAD50 expression with signature 3 activity across BRCA samples. For this signature, the mutation counts in both isolated and close are positively correlated with expression of considered genes

**Fig. 7:**
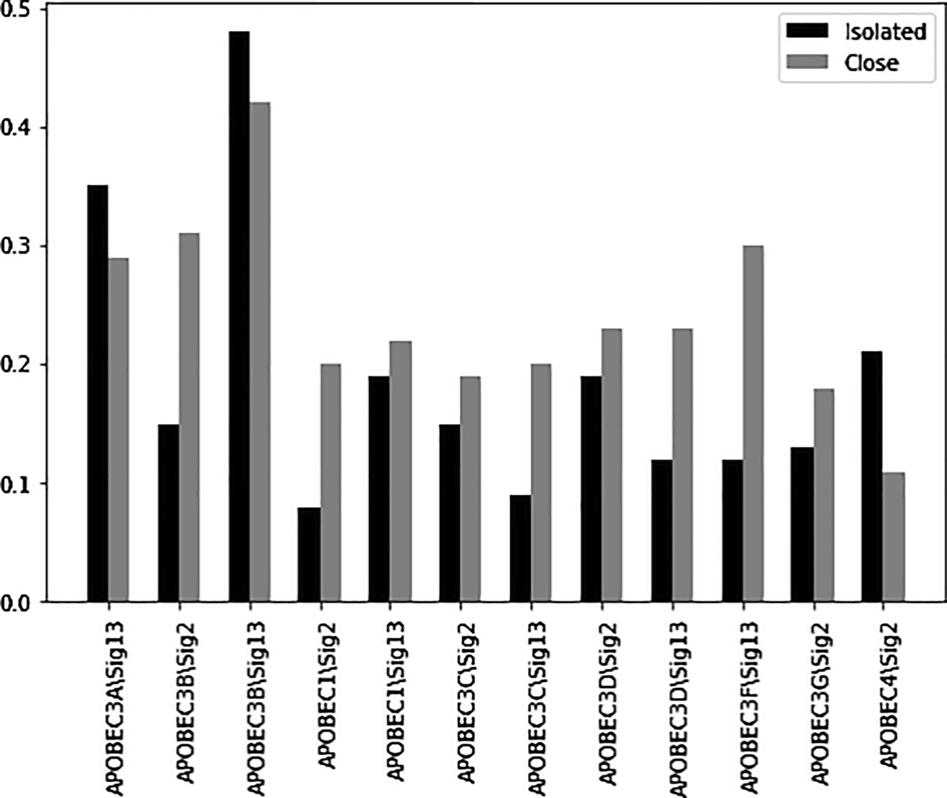
Spearman correlation comparison of the APOBEC3A, APOBEC3B, APOBEC1, APOBEC3C, APOBEC3D, APOBEC3F, APOBEC3G and APOBEC4 expression with signatures 2 and 13 activity across BRCA samples. For this signature, the mutation counts in both isolated and close are positively correlated with expression of considered genes

Also, the expression of ten other selected genes by proposed model, namely BRCA1, BRCA2, ATM, CHEK2, MRE11A, NBN, FANCA, PALB2, RAD51C, RAD50 has high Spearman correlation coefficient with activity of mutation signature 3. The Spearman correlation coefficients between the expressions of these genes with a signature 3 activity are shown in Fig. 6. Similarly, in a recent study [35] it was shown that homologous recombination deficiency (HRD) is associated with the signature 3 in breast cancer patients. Homologous recombination deficiency is the inability to repair double-strand breaks in human cells. Several genetic alterations causing HRD include somatic mutations of genes such as the selected 10 genes. The eight other selected genes by the Auto-HMM-LMF method for BRCA cancer cell lines are APOBEC3A, APOBEC3B, APOBEC1, APOBEC3C, APOBEC3D, APOBEC3F, APOBEC3G and APOBEC4 that belong to the APOBEC family. The Spearman correlation coefficients between the expression of these genes with 2 and 13 signature activities are shown in Fig. 7. A recent study [35] has conducted that APOBEC deamination of cytosine to uracil is thought to initiate mutations of signatures 2 and 13. Therefore, these results show that the genes selected for breast cancer are biologically essential, and the Auto-HMM-LMF method has been able to detect significant features for single nucleotide mutation data. In addition to increasing the accuracy of drug response prediction compared to other models, one of the most important advantages of the Auto-HMM-LMF algorithm is that this algorithm's running time is significantly lower than the running time of the other mentioned methods. Since this method is based on the selection of features, one of its limitations is that the results depend on the selected features. So, this method's results can be improved by using a more powerful feature selection approach. The following limitation of the method is that it was designed to solve the cold problem for a new cell line, while some of the proposed methods can also make predictions for the new drug or new pair of cell line-drug.

## Conclusion

In this study, we developed a feature selection-based method, Auto-HMM-LMF, to predict cancer cell lines’ response to drugs in the GDSC and CCLE datasets. For feature selection of gene expression and copy number variation data, two autoencoder networks are designed. For feature selection of single nucleotide mutation information, the novel approach based on the hidden Markov model (HMM) and multinomial mixture model (MMM) is applied. Auto-HMM-LMF shows better overall prediction performance than the state-of-the-art prediction methods. Also, by comparing to one of the most powerful feature selection methods, the EFS method, we showed that the performance of the predictive model based on selected features introduced in this paper is much better for drug response prediction. Also, we suggest that the proposed model can be useful in numerous therapeutic research areas, such as drug repositioning and personalized medicine. Finally, we found substantial evidence that the selected features and predicted responses by Auto-HMM-LMF have significant consistency with many previous studies.

## Supplementary Information

**Additional file 1.** The results of the correlation between some genes and signature activity in 14 cancer types with high Spearman correlation coefficients.

**Additional file 2.** The similarity matrices based on tissue type information corresponding to GDSC dataset.

**Additional file 3.** The similarity matrices based on tissue type information corresponding to CCLE dataset.

**Additional file 4.** The scatter plots of observed and predicted drug responses by the Auto-HMM-LMF model of 20 drugs in the CCLE dataset.

## Data Availability

The data and implementation are accessible from (https://github.com/emdadi/Auto-HMM-LMF).

## Abbreviations

GDSC: Genomics of drug sensitivity in cancer
CCLE: Cancer cell line encyclopedia
EFS: Ensemble feature selection
SRMF: Similarity regularized matrix factorization
CaDRReS: Cancer drug response prediction using a recommender system
DSPLMF: Drug sensitivity prediction based on logistic matrix factorization
COSMIC: Catalogue of somatic mutations in cancer
HMM: Hidden Markov model
MMM: Multinomial mixture model
RTK: Receptor tyrosine kinase
MMR: Mismatch repair
HRD: Homologous recombination deficiency
