# Supplementary material for: Auto-HMM-LMF: feature selection based method for prediction of drug response via autoencoder and hidden Markov model
Source: BMC Bioinformatics. 2021 Jan 28;22:33. doi: 10.1186/s12859-021-03974-3 (PMC7844991; doi:10.1186/s12859-021-03974-3)
Supplement: Supplementary file 4 — Additional file 4. The scatter plots of observed and predicted drug responses by the Auto-HMM-LMF model of 20 drugs in the CCLE dataset. [file 12859_2021_3974_MOESM4_ESM.pdf]

The scatter plots of 20 drugs in the CCLE dataset

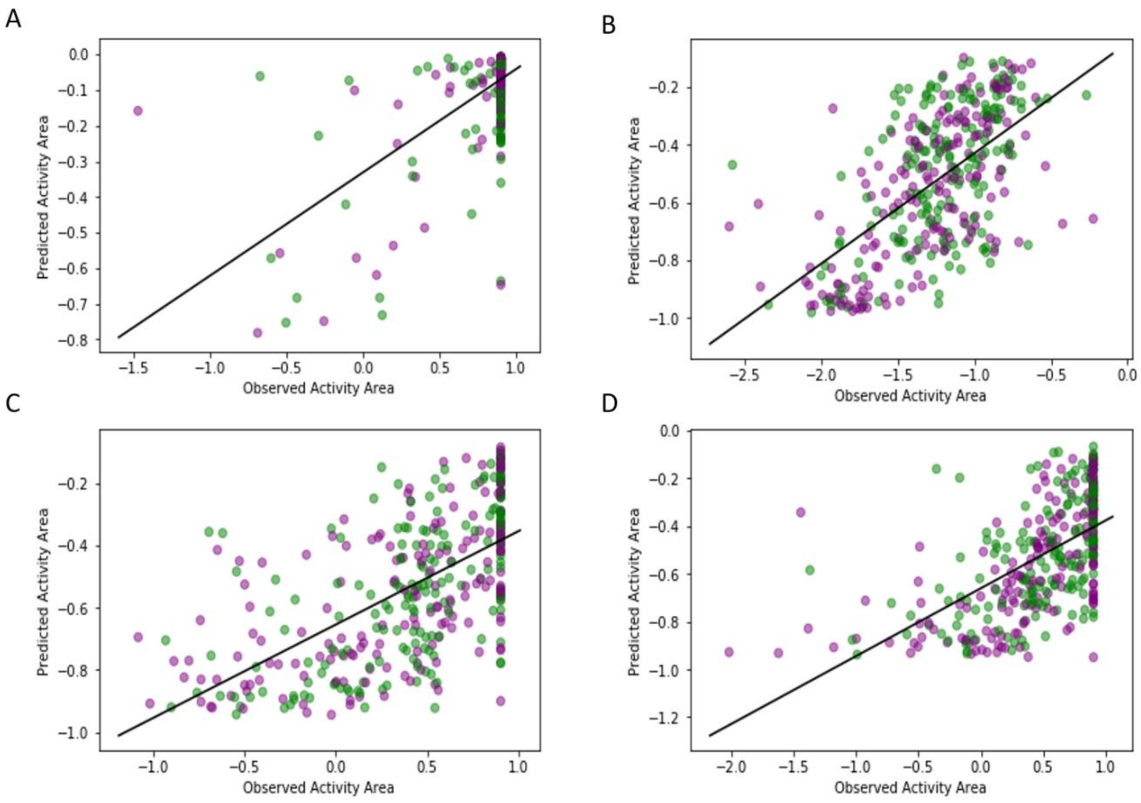

Figure 1: Correlations between observed and predicted activity areas for CCLE cell lines across 4 drugs using AutoHMM-LMF method. The green points represent the observed values and purple points represent the predicted values by Auto-HMM-LMF method. **(A)** The scatter plot of observed and predicted drug responses for PLX4720 (Pearson correlation coefficient=0.62). **(B)** The scatter plot of observed and predicted drug responses for $Panobinostat$ (Pearson correlation coefficient=0.60). (**C**) The scatter plot of observed and predicted drug responses for RAF265 (Pearson correlation coefficient=0.61). **(D)** The scatter plot of observed and predicted drug responses for TAE684 (Pearson correlation coefficient=0.59).

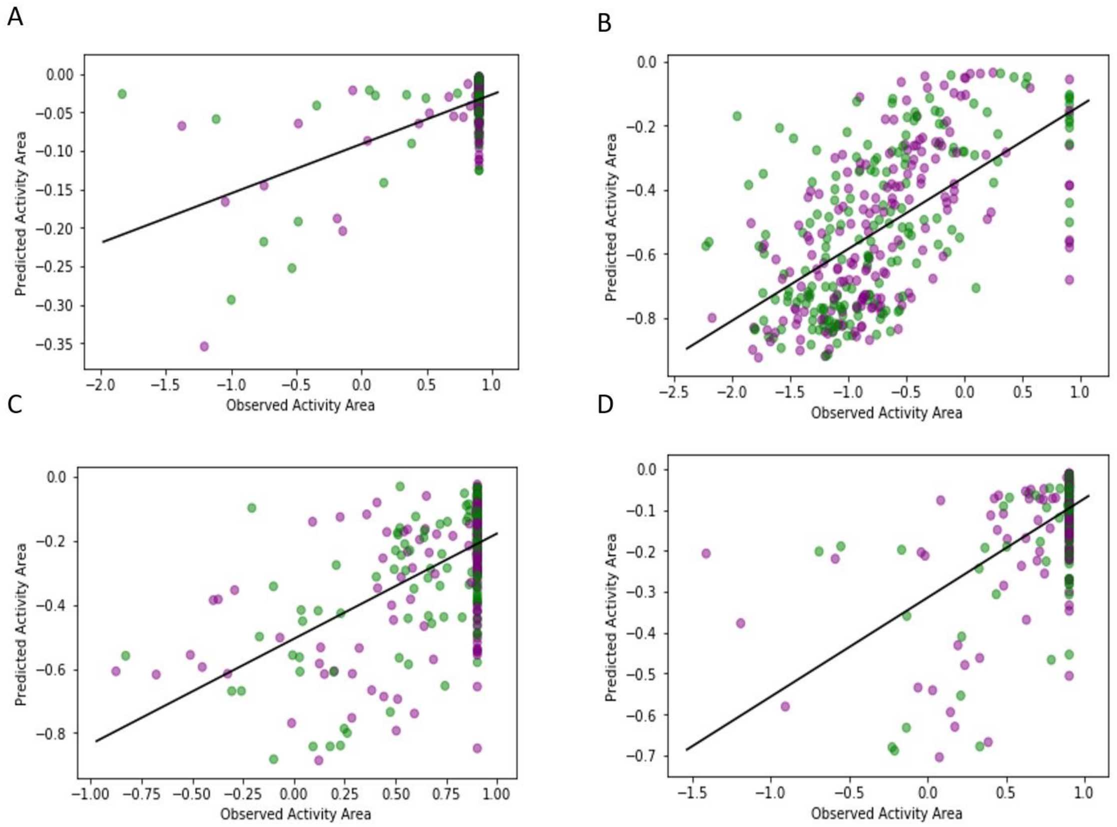

Figure 2: Correlations between observed and predicted activity areas for CCLE cell lines across 4 drugs using AutoHMM-LMF method. The green points represent the observed values and purple points represent the predicted values by Auto-HMM-LMF method. **(A)** The scatter plot of observed and predicted drug responses for $LBW242$ (Pearson correlation coefficient=0.60). **(B)** The scatter plot of observed and predicted drug responses for $17 - AAG$ (Pearson correlation coefficient=0.59). **(C)** The scatter plot of observed and predicted drug responses for $AZD0530$ (Pearson correlation coefficient=0.57). **(D)** The scatter plot of observed and predicted drug responses for $Erlotinib$ (Pearson correlation coefficient=0.58).

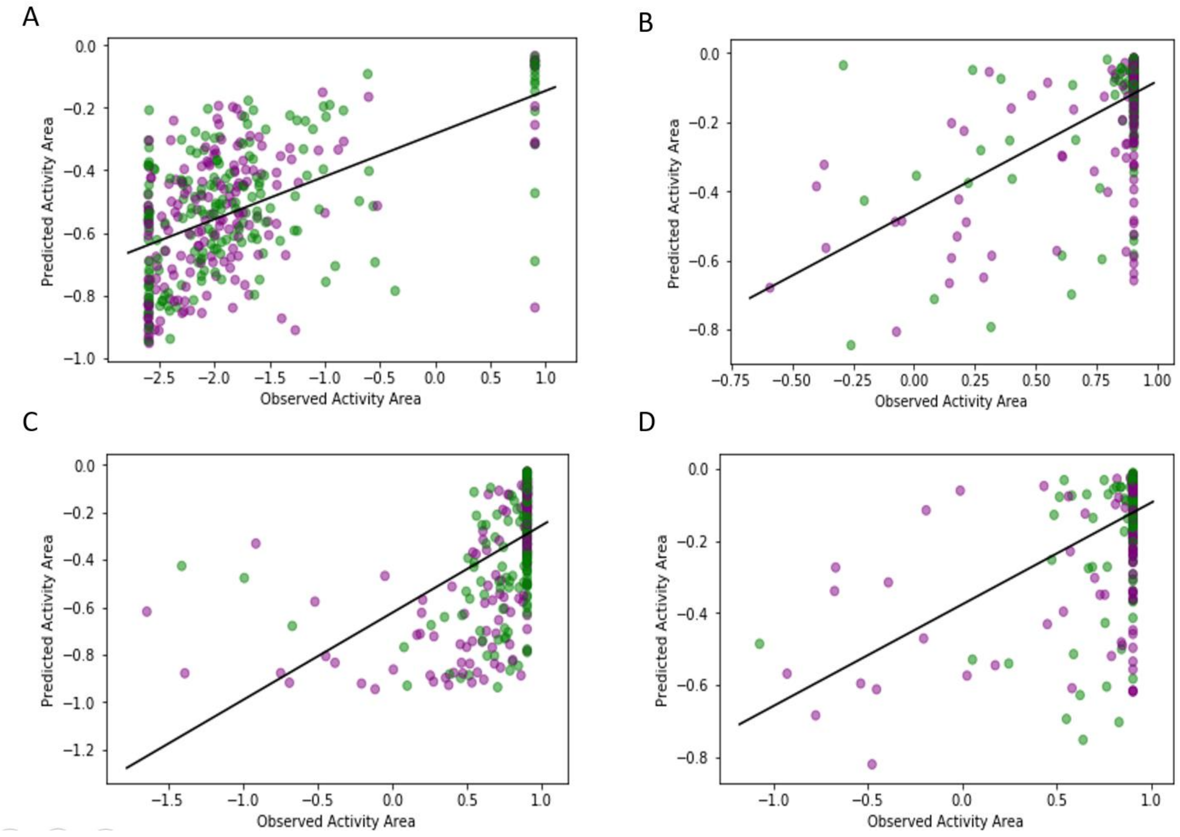

Figure 3: Correlations between observed and predicted activity areas for CCLE cell lines across 4 drugs using AutoHMM-LMF method. The green points represent the observed values and purple points represent the predicted values by Auto-HMM-LMF method. **(A)** The scatter plot of observed and predicted drug responses for $Paclitaxel$ (Pearson correlation coefficient=0.56). **(B)** The scatter plot of observed and predicted drug responses for PD−0332991 (Pearson correlation coefficient=0.56). **(C)** The scatter plot of observed and predicted drug responses for $Crizotinib$ (Pearson correlation coefficient=0.51). **(D)** The scatter plot of observed and predicted drug responses for L − 685458 (Pearson correlation coefficient=0.50).

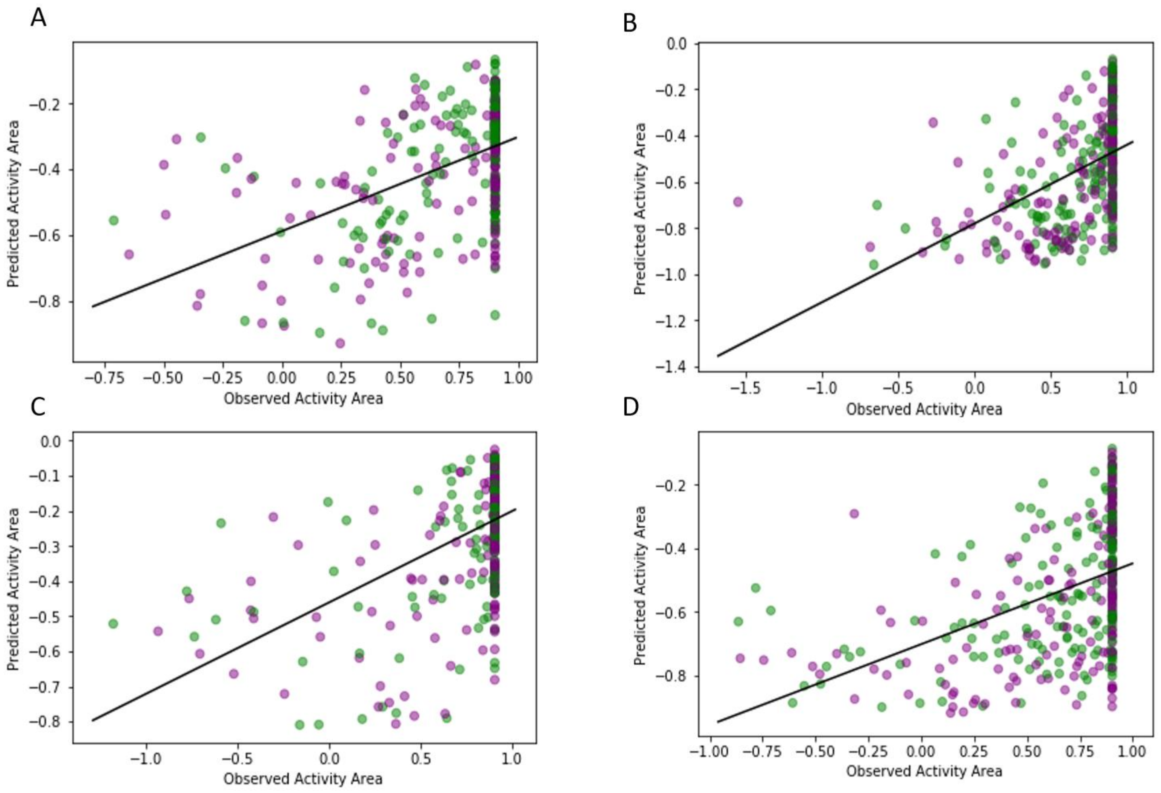

Figure 4: Correlations between observed and predicted activity areas for CCLE cell lines across 4 drugs using AutoHMM-LMF method. The green points represent the observed values and purple points represent the predicted values by Auto-HMM-LMF method. **(A)** The scatter plot of observed and predicted drug responses for $vandetanib$ (Pearson correlation coefficient=0.49). **(B)** The scatter plot of observed and predicted drug responses for TKI258 (Pearson correlation coefficient=0.48). **(C)** The scatter plot of observed and predicted drug responses for $lapatinib$ (Pearson correlation coefficient=0.51). **(D)** The scatter plot of observed and predicted drug responses for AEW541 (Pearson correlation coefficient=0.46).

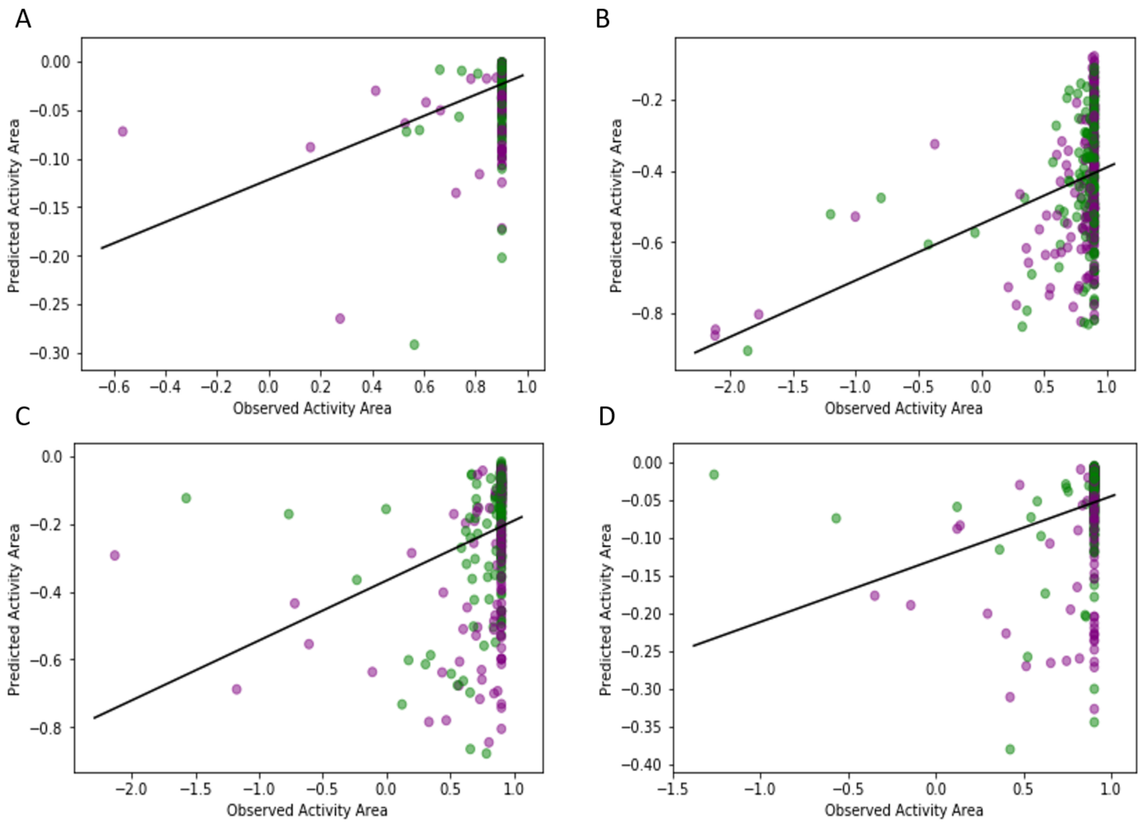

Figure 5: Correlations between observed and predicted activity areas for CCLE cell lines across 4 drugs using AutoHMM-LMF method. The green points represent the observed values and purple points represent the predicted values by Auto-HMM-LMF method. **(A)** The scatter plot of observed and predicted drug responses for $nutlin - 3$ (Pearson correlation coefficient=0.33). **(B)** The scatter plot of observed and predicted drug responses for $nilotinib$ (Pearson correlation coefficient=0.32). **(C)** The scatter plot of observed and predicted drug responses for $sorafenib$ (Pearson correlation coefficient=0.28). **(D)** The scatter plot of observed and predicted drug responses for $PHA - 665752$ (Pearson correlation coefficient=0.24).
